# The Treatment of Epilepsy

**Published:** 1847-12

**Authors:** 


*The Treatment of Epilepsy*, by Professors Rostan, Velpeau, Williams, &c.—By reference to the letter of the Editor, in the * last No. of this Journal, it will be seen at thhe had not at its date received communications from the above named gentlemen, on the subject upon which they had been consulted. He now briefly gives the treatment recommended by them in the case alluded to:—

1st. Take for drink the infusion of the flowers of the peach tree, or the leaves of the laurel.

2d. Take three times a week a bath with the infusion of the linden tree, or of the leaves of the laurel, and of the temperature of 98°, while cold water is poured upon the head—the patient remaining in it four or five hours.

3d. In the interval between the bath, the patient will occasionally be placed standing in a foot-bath at the temperature of 104°, and water at 78° be poured upon the head.

4th. Take of the powdered *root* of belladonna, one grain each day for the first week, and increase one gr. each week, watching carefully the effects of this article upon the system—diminishing, augmenting or suspending it, according to its action.

5th. Let the patient be purged at least once a week.

(Signed,) Rostan.
Paris, 14th August. Velpeau.

M. Leuret, physician in chief to the Bicetre Hospital, was also consulted He agreed with the treatment just related, and laid great stress upon *exercise to fatigue*, and also recommended to arrest the premonitory symptoms of attack, a large dose of opium and musk.

Prof. Williams's letter is as follows:

London, Holles St., Aug. 25, 1847.

The chief indication in the treatment of these cases is obviously to endeavor as much as possible to equalize and strengthen the circulation, whilst all causes of occasional embarrassment or excitement are carefully avoided or counteracted. The treatment of

---

* See last No. of Buffalo Medical Journal.

each individual case will require a very careful examination into the state of all the functions, and the application of fitting measures to correct any that may be in disorder. The functions which I have found most frequently erring (even when not mentioned by the patient) are those of the heart and kidneys. Palpitation often precedes the attack without being obvious to the patient; and to prevent this I have found hydrocyanic acid or digitalis, in doses gradually increased, very serviceable remedies; and they may be combined with tonics or otherwise, according to the condition of the system. An unhealthy state of urine, manifested by either scantiness or albuminons impregnation, I have discovered in several cases, and have corrected by a large blister to the loins, followed by a free and long continued use of the expressed juice of Taraxacum (or Extract prepared without heat) together with bi-carbonate of potash or other diuretic salt. In addition to these means, others calculated to improve the general health and tone of the circulation —much open air—regular moderate exercise, careful diet. &c,— should not be overlooked.

The prognosis is extremely varied, and cannot be stated without a very minute knowledge and some experience of the case. I have known many cases recover partially—some entirely: in others no improvement took place, but ultimately gradual lapse into altered structure of the encephalon. A lady, the mother of one case now under my care, was subject to occasional fits from puberty until her second confinement, which happened about 14 years ago, since which she has had none. Her daughter has also had several since puberty, but she has had only one in the last 12 months, during which she has three times a day taken gr. iss. Zinci Sulphat. with gr. xij. Ext. Flor. Taraxaci (prepared without heat).

The above are the best hints which I can offer, without the advantage of seeing the patient; and remain,

Yours, faithfully,

C. J. B. Williams.

[*Southern Med. and Surg. Journal.*

